# Quantitative analysis with load–displacement ratio measured via digital arthrometer in the diagnostic evaluation of chronic ankle instability: a cross-sectional study

**DOI:** 10.1186/s13018-022-03177-3

**Published:** 2022-05-23

**Authors:** Yungu Chen, Shengxuan Cao, Chen Wang, Xin Ma, Xu Wang

**Affiliations:** Department of Orthopedics, Huashan Hospital, Fudan University, Jingan District, Shanghai, China

**Keywords:** Chronic ankle instability, Arthrometer, Diagnostic accuracy, Sensitivity, Specificity, Load–displacement ratio

## Abstract

**Background:**

Arthrometry has been introduced to evaluate the laxity of ankle joint in recent years. However, its role in the diagnosis of chronic ankle instability is still debatable. Therefore, we assessed the diagnostic accuracy of a digital arthrometer in terms of sensitivity and specificity.

**Methods:**

According to the inclusion and exclusion criteria proposed by the International Ankle Consortium, we recruited 160 uninjured ankles (control group) and 153 ankles with chronic ankle instability (CAI group). Ankle laxity was quantitively measured by a validated digital arthrometer. Data of loading force and joint displacement were recorded in a continuous manner. Differences between the control and CAI groups were compared using 2-tailed independent t test. A receiver operating characteristic curve analysis was used to calculate area under a curve, sensitivity and specificity.

**Results:**

Load–displacement curves of the two groups were depicted. Differences of joint displacement between the control and CAI groups were compared at 30, 45, 60, 75, 90, 105 and 120 N, which were all of statistical significance (all *p* < 0.001) with the largest effect size at 90 N. Statistical significance was found in the differences between the two groups in load–displacement ratio at 10–120 N, 10–40 N, 40–80 N and 80–120 N (all *p* < 0.001), with the largest effect size at 10–40 N. Load–displacement ratio at the interval of 10–40 N had the highest area under a curve (0.9226), with sensitivity and specificity of 0.804 and 0.863, respectively, when the cutoff point was 0.1582 mm/N.

**Conclusion:**

The digital arthrometer measurement could quantitively analyze the ankle laxity with high diagnostic accuracy. The load–displacement ratio would be a reliable and promising approach for chronic ankle instability diagnosis.

*Level of evidence* level II.

## Background

Ankle sprain is one of the most frequently encountered traumatic injuries in clinical settings [1]. It is commonly caused by a sudden force of plantar flexion, inversion or internal rotation which can injure the lateral ankle ligaments [2]. Lateral ankle ligaments comprise anterior talofibular ligament (ATFL), calcaneofibular ligament (CFL) and posterior talofibular ligament (PTFL) [3]. Nearly 20% of patients with ankle sprain develop residual symptoms, including pain, swelling, recurrent ankle sprains, giving way and feeling of instability; these symptoms are collectively known as chronic ankle instability (CAI) [4].

Despite the numerous studies of CAI, there is still no consensus about the gold standard of CAI diagnosis. The proper diagnosis of CAI is a difficult task because the association between inversion trauma history and ligament injury is still uncertain [5]. Manual stress tests, including anterior drawer, anterolateral drawer and talar tilt test, have been widely utilized in clinical practice. Nevertheless, the manual stress tests still have limited diagnostic accuracy due to their subjective and qualitative properties [6]. Li et al. [7] reported that the specificity of manual anterior drawer test (ADT) was as high as 1, but the sensitivity was only 0.053 in junior doctors and 0.395 in senior doctors. This reflects manual tests’ nature of subjectivity. Thus, quantitative analysis of talocrural joint laxity is necessary to improve the accuracy and precision of the diagnostic test.

Recent literature showed that instrumented stress testing could quantify the laxity of talocrural joint; however, only few studies focused on the validity of arthrometers in terms of specificity and sensitivity. Croy et al. [8] found that the sensitivity and specificity of ADT were 0.83 (95% confidence interval [CI]: 0.64–0.93) and 0.40 (95% CI: 0.27–0.56), respectively, when comparing 20 controls and 66 ankle-injured. Lohrer et al. [6] recruited 41 patients that were primarily diagnosed with functional ankle instability (FAI) and divided them into 2 groups according to the mechanical stability of their ankles. They found that arthrometer ADT had sensitivity of 0.81 and specificity of 0.93 in differentiating mechanically stable and unstable ankles. The variety of results could be explained by the high heterogenicity regarding studied population, sample sizes, selection criteria and arthrometric devices [9]. Therefore, studies with standardized method design and an adequate sample size are needed to evaluate the diagnostic accuracy of instrumented stress testing in CAI.

The purpose of this study was to design a cross-sectional study with standardized inclusion/exclusion criteria and an adequate sample size to quantitively assess the diagnostic accuracy of ankle arthrometers. In this study, we were to compare different diagnostic standards and calculate the diagnostic accuracy of each standard. Our hypothesis was that arthrometers would display an excellent diagnostic accuracy on CAI.

## Methods

A cross-sectional study was conducted to quantitatively investigate the difference in ankle joint laxity between the CAI and control groups from October 2020 to September 2021. This study was approved by the local ethics committee. All participants provided written informed consent.

### Selection criteria

We made the inclusion and exclusion criteria according to the international Ankle Consortium [10]. For the CAI group, all the following inclusion criteria had to be met: (1) a history of at least 1 significant ankle sprain with the initial sprain having occurred at least 12 months prior to the recruitment; (2) associated inflammatory symptoms (pain, swelling, etc.); (3) at least one interrupted day of desired physical activity; (4) a history of at least 2 episodes of sprains and/or “feelings of instability” and/or “giving way” in the 6 months prior to the study enrolment; (5) cumberland ankle instability tool (CAIT) [11] scores lower than 24. For the control group, the inclusion criteria were: (1) no history of ankle injury, instability or surgery; (2) normal ankle range of motion and muscle strength; (3) CAIT scores of 29 or 30 [12]. Overall exclusion criteria were: (1) age not within 18–50 years; (2) a history of surgeries to the musculoskeletal structures in either lower extremity; (3) a history of fracture in either lower extremity requiring realignment; (4) a history of acute injury to the lower extremity within 3 months before the enrollment. Subjects who had neuromuscular disorders, obesity (BMI > 30) or intolerance of force applied by the arthrometer during instrumented testing were also excluded. People who reported a history of ankle sprain but had no residual symptoms were defined as copers, and they were not included in this study.

### Sample size calculation

Sample size was calculated by the formula designed for quantitative variables in cross-sectional studies:

$${\text{Sample}}\;{\text{size}} = ({\text{Z}}_{{{1} - \alpha /{2}}} )^{{2}} \left( {{\text{SD}}} \right)^{{2}} /{\text{d}}^{{2}} \;\left[ {{13}} \right]$$

Z_1−*α*/2_ = Standard normal variate, which is 1.96 at 5% type 1 error (*p* < 0.05).

SD = Standard deviation of variable. Value can be taken from previously done study.

d = Absolute error or precision decided by researchers.

SD was 5.66 mm according to a previous study [14], and d was set as 1 mm. Therefore, the sample size was calculated to be at least 123 ankles in each group.

### Participants

**Fig. 1:**
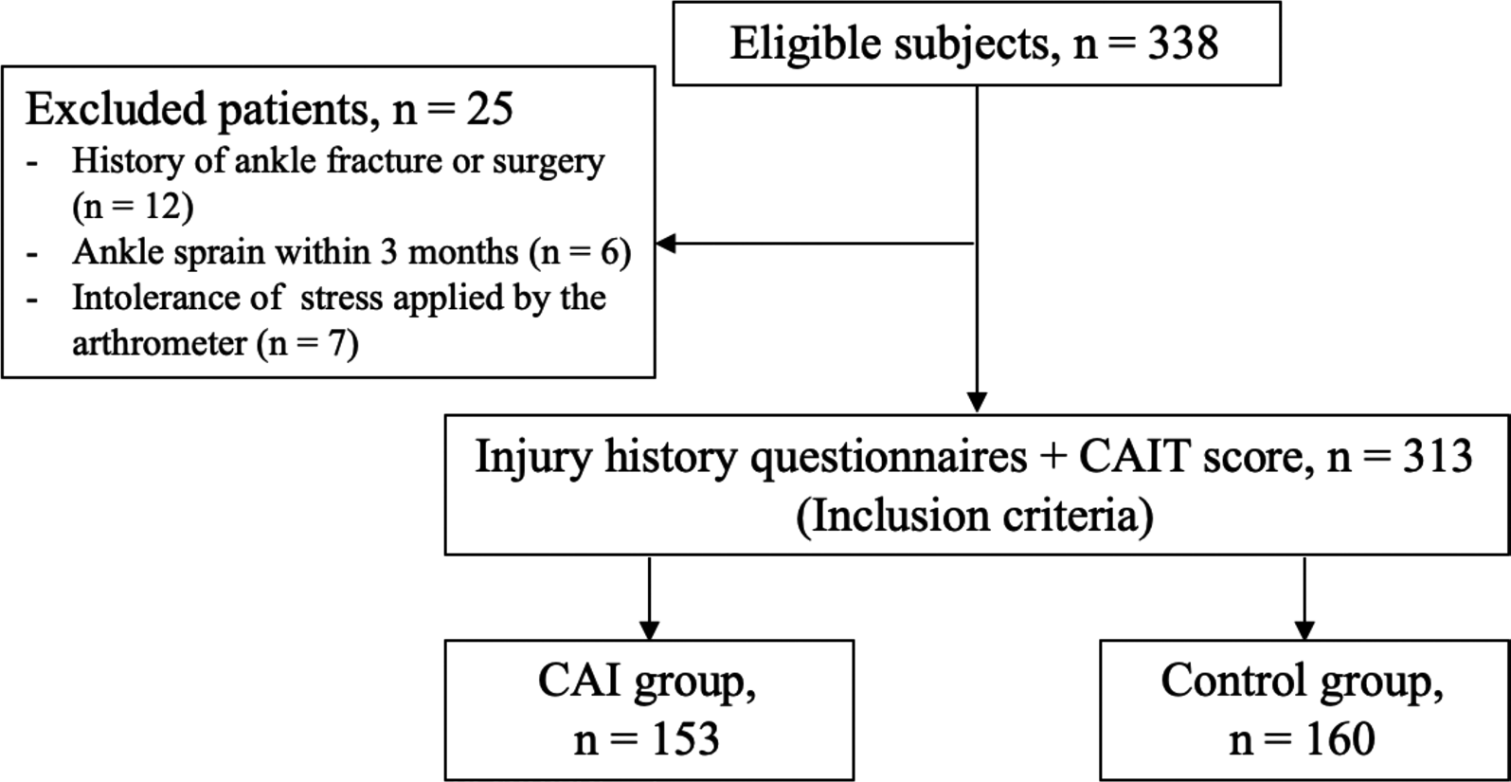
Flow diagram to demonstrate the recruitment procedure of the tested groups. *CAIT* Cumberland ankle instability tool; *CAI* chronic ankle instability

A total of 338 subjects consented to the participation in the study and underwent the testing procedure. Among them, 25 were excluded: 12 had a history of ankle fracture or surgery, 6 experienced at least one episode of ankle sprain within 3 months prior to the enrollment, and 7 could not tolerate the stress applied by the arthrometer. At last, 313 subjects were included in the study. In total, there were 160 subjects assigned to the control group and 153 to the CAI group according to the inclusion and exclusion criteria (Fig. 1). No treatments were applied during the time of instrumented stress testing.

### Experimental procedure

**Fig. 2:**
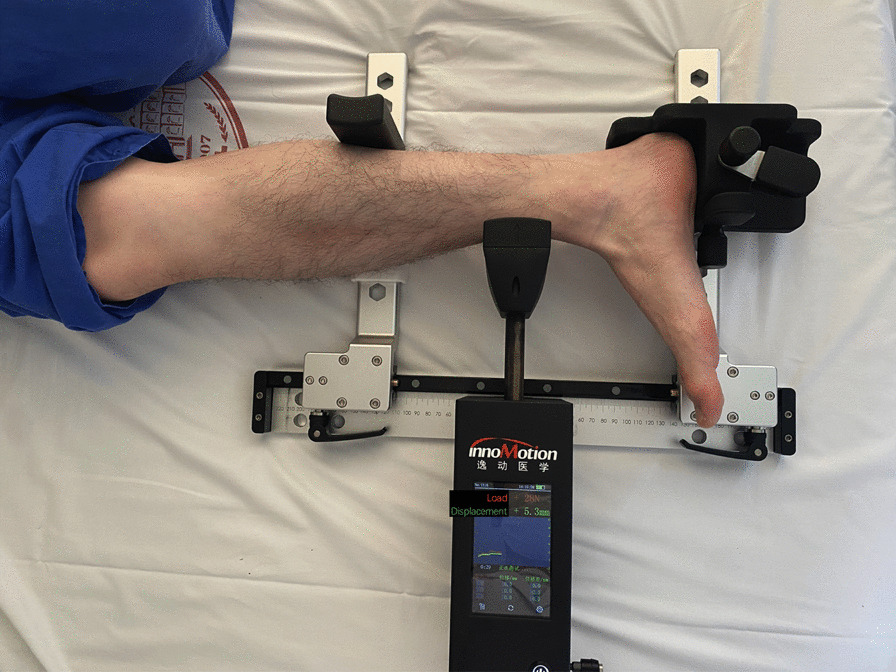
Instrumented anterior drawer test by Ligs Digital Arthrometer. The force is applied against the anterior tibia, while the heel and the calf were locked for counterforce

**Fig. 3:**
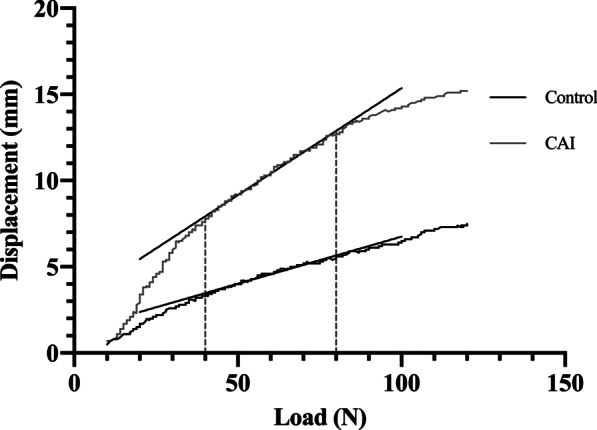
Two typical load–displacement curves. The black one is selected from the control group, and the gray one is selected from the CAI group. Linear regression’s slopes were utilized to calculate load–displacement ratios at different load intervals. Notice that the slope of the CAI is steeper than the control, indicating greater laxity. *CAI* chronic ankle instability

Instrumented ADT was performed with Ligs Digital Arthrometer (Innomotion Inc., China, Fig. 2), which required no radiographic assistance to objectively quantify ADT. The motor unit of this device gradually pulled the anterior tibia posteriorly with respect to a fixed calf and heel (3 N/s, maximum force 120 N), and loading force and joint displacement were recorded continuously by the sensor unit. The record started when the load exceeded 10 N to reduce the influence of calf musculature. The data were later transferred to a laptop for further analysis. Some studies indicated that the ratio of load and displacement could serve as a dynamic reference standard to represent ankle joint laxity. [6, 15] From the arthrometer-produced load–displacement curve, load–displacement ratios (LDRs) at different load intervals were calculated (Fig. 3). The load was accurate to 1 N, and the displacement was accurate to 0.1 mm.

The data were analyzed for only 1 ankle per individual. For individuals in the control group, the side to be included in the group was randomly selected. For individuals in the CAI group, the self-described “worse” side was chosen. On the tested side, the procedure was performed 3 times. Ankle arthrometer calculations were based on the average values of 3 consecutive measurements. Reliability of the arthrometer in our study was also validated. The intraclass correlation coefficient (ICC_2,1_) of a single measure was 0.897 (95% CI, 0.227–0.969), and the ICC_2,2_ was 0.963 (95% CI, 0.469–0.989) when using the average of 3 measures, indicating an excellent test–retest reliability. Inter-tester reliability was evaluated by taking measurement of the same ankle made by 2 independent examiners, and the ICC_2,1_ was 0.949, indicating an excellent inter-tester reliability.

### Statistical analysis

Statistical analysis was performed by IBM SPSS Statistics 24.0. In order to compare the control and CAI groups, static reference standards were joint displacement values measured at fixed loads of 30 N, 45 N, 60 N, 75 N, 90 N, 105 N and 120 N, while dynamic reference standards were load–displacement ratios at the load intervals of 10–120 N, 10–40 N, 40–80 N and 80–120 N. Differences were calculated using 2-tailed independent t test unless specified otherwise. Effect size was calculated by the Cohen’s D, where the strength of the effect size was determined as small (0.20), medium (0.50) or large (0.80) [16]. A receiver operating characteristic curve (ROC) analysis was used to calculate the cutoff values which discriminated between the control and CAI groups. Area under a ROC curve (AUC) was also calculated to inspect the diagnostic accuracy of each reference standard, where larger AUC indicated higher diagnostic accuracy [17]. Sensitivity and specificity were also calculated. The level of significance was set a priori at *p* < 0.05.

## Results

### Demographics

Subjects’ demographic characteristics in comparisons between the control and CAI groups are shown in Table 1. There were significant differences between the two groups in BMI (*p* = 0.047). This might be due to a larger proportion of male in the CAI group (83/153, 54.2%) than the control group (73/160, 45.6%). The CAI group’s CAIT scores were significantly lower than the control group (*p* < 0.001).

**Table 1:** Demographic characteristics of subjects by group^*^

Variable*	Control (*n* = 160)	CAI (*n* = 153)	*p* value
Male sex^**^ (%)	73 (45.6%)	83 (54.2%)	0.094
Age, y	30.49 $\pm$ 8.11	30.84 $\pm$ 9.43	0.746
BMI	22.11 $\pm$ 3.01	22.76 $\pm$ 2.34	**0.047**
Total sprains, *n*	NA	3.65 $\pm$ 1.82	
Tested side, left^**^ (%)	78 (48.8%)	79 (51.6%)	0.317
Time since last sprain, mo	NA	17.94 $\pm$ 15.87	
CAIT score	29.93 $\pm$ 0.25	19.75 $\pm$ 5.78	**< 0.001**

*CAI* chronic ankle instability, *BMI* body mass index, *NA* not available, *CAIT* cumberland ankle instability tool, *SD* standard deviation

^*^Values are presented as mean $\pm$ SD unless specified otherwise; the level of significance is set a priori at *p* < 0.05

^**^Statistical differences are calculated by Pearson *χ*^2^ test

### Reference standard comparisons

**Fig. 4:**
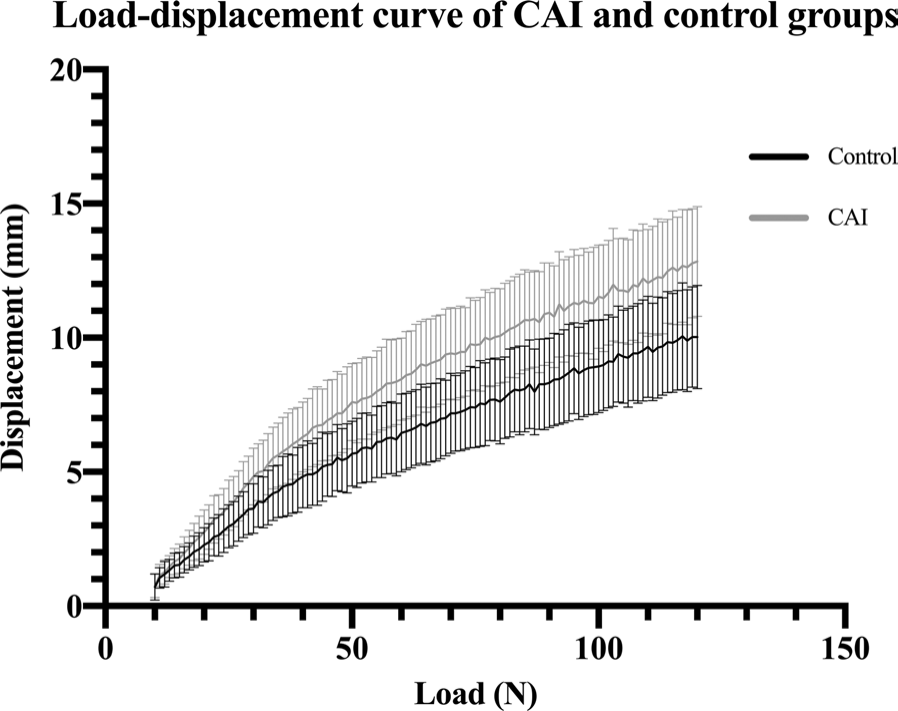
Load–displacement curves of the CAI and control groups. Each point of the curve is presented as mean $\pm$ SD. *CAI* chronic ankle instability; *SD* standard deviation

By depicting each individual load–displacement curve, the overall load–displacement curves of the control and CAI groups were derived (Fig. 4). For static reference standards, differences of displacement between the control and CAI groups at 30, 45, 60, 75, 90, 105 and 120 N were all of statistical significance (all *p* < 0.001) with the largest effect size at the load of 90 N (effect size = 1.62, Table 2). For dynamic reference standards, LDRs and corresponding R square of the linear regression were calculated at the intervals of 10–120 N, 10–40 N, 40–80 N and 80–120 N (Table 3). Comparing the control and CAI group, statistical significance was found in the differences between the two groups in LDRs at 10–120 N, 10–40 N, 40–80 N and 80–120 N (all *p* < 0.001). with the largest effect size at 10–40 N (effect size = 1.85).

**Table 2:** Comparisons of displacement of the control and CAI groups by force^*^

Load	Control (*n* = 160)	CAI (*n* = 153)	*p* value	Effect size
30 N	3.636 ± 0.875	4.794 ± 0.998	**< 0.001**	1.23
45 N	5.185 ± 1.228	6.931 ± 1.243	**< 0.001**	1.41
60 N	6.300 ± 1.384	8.511 ± 1.422	**< 0.001**	1.58
75 N	7.348 ± 1.537	9.837 ± 1.566	**< 0.001**	1.60
90 N	8.250 ± 1.650	10.939 ± 1.669	**< 0.001**	1.62
105 N	9.166 ± 1.791	11.989 ± 1.765	**< 0.001**	1.59
120 N	9.937 ± 1.906	12.865 ± 1.873	**< 0.001**	1.55

*CAI* chronic ankle instability, *SD* standard deviation

^*^Values are presented as mean $\pm$ SD; the level of significance is set a priori at *p* < 0.05

**Table 3:** Load–displacement ratios (LDRs) of different intervals of the load–displacement curve

	Control (*n* = 160)		CAI (*n* = 153)		*p* value	Effect size
	LDR (mm/N)	R^2^	LDR (mm/N)	R^2^		
LDR at 10–120 N	0.0777 ± 0.0176	0.954 ± 0.025	0.1068 ± 0.0215	0.926 ± 0.042	**< 0.001**	1.48
LDR at 10–40 N	0.1160 ± 0.0360	0.969 ± 0.025	0.1924 ± 0.0459	0.962 ± 0.031	**< 0.001**	1.85
LDR at 40–80 N	0.0735 ± 0.0211	0.984 ± 0.021	0.0978 ± 0.0280	0.971 ± 0.024	**< 0.001**	0.98
LDR at 80–120 N	0.0571 ± 0.0139	0.986 ± 0.010	0.0667 ± 0.0186	0.985 ± 0.009	**< 0.001**	0.58

*CAI* chronic ankle instability, *LDR*: Load–displacement ratio, *SD* standard deviation

Values are presented as mean $\pm$ SD. LDRs are calculated by linear regression. The level of significance is set a priori at *p* < 0.05

### Diagnostic accuracy

**Fig. 5:**
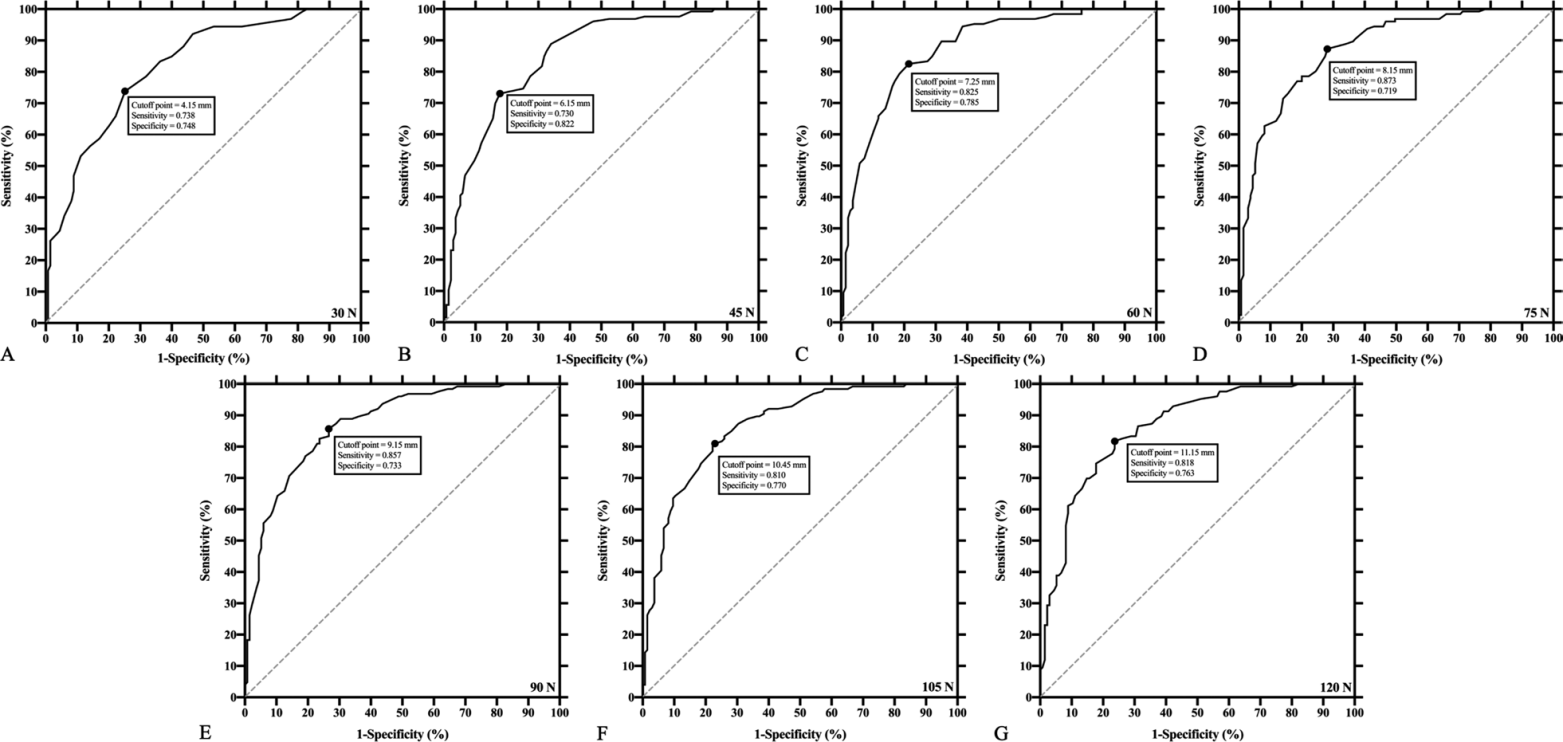
ROCs of displacement values at different loads applied by the arthrometer. **A** load = 30 N; **B** load = 45 N; **C** load = 60 N; **D** load = 75 N; **E** load = 90 N; **F** load = 105 N; **G** load = 120 N. *ROC* receiver operating characteristic curve

**Fig. 6:**
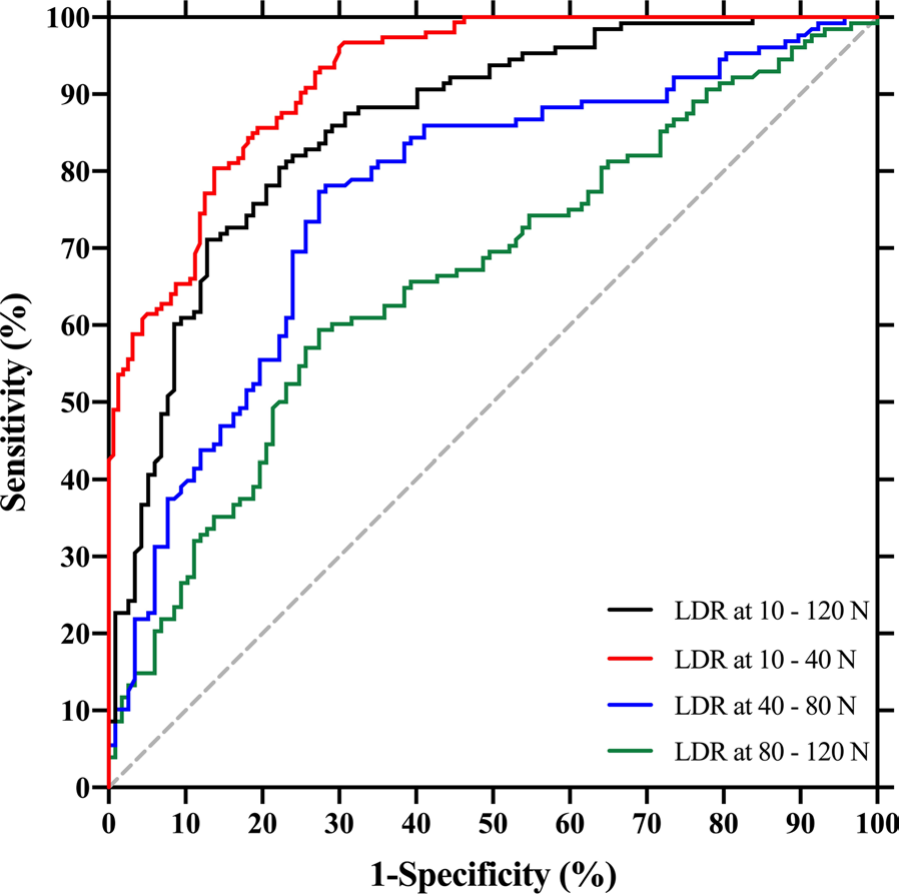
ROC of LDRs at different load intervals. *ROC* receiver operating characteristic curve, *LDR* load–displacement ratio

A ROC analysis was used to calculate the diagnostic accuracy of each reference standard (Table 4). Displacement presented with the highest AUC (0.876 [95% CI, 0.834–0.917]) at the load of 75 N when used for the diagnosis of CAI. Cutoff value set at 8.15 mm, the sensitivity and specificity were 0.873 (95% CI, 0.804–0.920) and 0.719 (95% CI, 0.637–0.788), respectively, at 75 N. LDR at the interval of 10–40 N had the highest AUC (0.9226 [95% CI, 0.8955–0.9497]), with the sensitivity and specificity of 0.804 (95% CI, 0.734–0.859) and 0.863 (95% CI, 0.801–0.907) when the cutoff point was 0.1582 mm/N. ROC curves of displacement values and LDRs were pictured in Figs. 5 and 6, respectively.

**Table 4:** Receiver operating characteristic curve (ROC) analysis of displacement values and LDRs

	AUC (95% CI)	Cutoff value	Sensitivity (95% CI)	Specificity (95% CI)
Displacement at		(mm)		
30 N	0.816 (0.765–0.867)	4.15	0.738 (0.655–0.807)	0.748 (0.669–0.814)
45 N	0.849 (0.802–0.895)	6.15	0.730 (0.647–0.800)	0.822 (0.749–0.878)
60 N	0.875 (0.834–0.917)	7.25	0.825 (0.750–0.882)	0.785 (0.709–0.846)
75 N	0.876 (0.834–0.917)	8.15	0.873 (0.804–0.920)	0.719 (0.637–0.788)
90 N	0.874 (0.832–0.915)	9.15	0.857 (0.786–0.908)	0.733 (0.653–0.801)
105 N	0.867 (0.824–0.910)	10.45	0.810 (0.732–0.869)	0.770 (0.693–0.833)
120 N	0.861 (0.817–0.905)	11.15	0.818 (0.741–0.875)	0.763 (0.685–0.827)
LDR at the interval of		(mm/N)		
10–120 N	0.860 (0.814–0.906)	0.0905	0.805 (0.728–0.864)	0.778 (0.694–0.844)
10–40 N	0.923 (0.896–0.950)	0.1582	0.804 (0.734–0.859)	0.863 (0.801–0.907)
40–80 N	0.766 (0.706–0.826)	0.0784	0.773 (0.694–0.837)	0.727 (0.640–0.799)
80–120 N	0.664 (0.596–0.731)	0.0633	0.594 (0.507–0.675)	0.727 (0.640–0.799)

*ROC* receiver operating characteristic curve, *LDR* Load–displacement ratio, *AUC* area under a ROC curve, *CI* confidence interval

## Discussion

In this study, we demonstrated that arthrometers present with good to high diagnostic accuracy, with sensitivity and specificity of 0.804 and 0.863, respectively. Dynamic measurement may have an advantage over static measurement in diagnosing CAI (Table 4). Load–displacement curves reflected the difference in laxity between CAI and normal ankles. At the lower region (10–40 N), the curve of the CAI group deviated from that of the control group with a steeper slope, which represented the anterior translation of talus [6]. Meanwhile, at the upper region (40–120 N) slopes of the control and CAI curves had a tendency to parallel to each other and both become less steeper, indicating that the talus was being translated to the end position and displacement was due to stiffer soft tissues that encompassed the ankle (Fig. 4).

Researchers tended to apply a force to a high magnitude with arthrometers, ranging from 125 to 200 N [6, 14, 18, 19]. However, this could be accompanied by significant discomfort felt by participants. In regard to static measurement in this study, a force of 90 N presented with the greatest effect size to differentiate the CAI and control groups, while a force of 75 N exhibited the largest AUC among all static reference standards (Tables 2 and 4). Meanwhile, the dynamic measurement indicated that a dynamically increasing force from 10 to 40 N had the highest effect size and the largest AUC (Tables 3 and 4). This indicates that a relatively low magnitude of force is adequate to discriminate CAI from uninjured ankles. Arthrometers have the best diagnostic accuracy (sensitivity = 0.804, specificity = 0.863) when using LDR at 10–40 N. Although the diagnostic accuracy of static reference standard is lower than dynamic reference standard, it may still be appropriate to diagnose CAI with arthrometers with a static applied force of 75 N, because the sensitivity and specificity (0.873 and 0.719, respectively) are acceptable for fast screening and testing in a clinical setting.

Nauck et al. [19] and Lohrer et al. [6] demonstrated that load–displacement curves served a good role in differentiation between ankle stable and unstable groups with arthrometers. In their studies, the slope between 40 and 60 N was used to present the stiffness of ankle joints. However, we found that LDR between 10 and 40 N had higher effect size and AUC, indicating better differentiating and diagnostic values, according to the load–displacement curves depicted in this study (Fig. 4). The use of LDR between 40 and 60 N was concluded from cadaver studies [20, 21], while our findings were based on an in vivo setting. Different fixation techniques in cadaver studies could alter kinematics and degrees of freedom of ankle joints when compared to an in vivo setting. Also, the properties of surrounding soft tissue may be altered when dissecting skin and capsula to approach the ankle ligaments in cadaver specimen, and thereby cause differences between cadaver and in vivo measurements [22]. Reviewing literature that used LDR as a reference standard, only Lohrer et al. [6] reported sensitivity and specificity (0.81 and 0.93, respectively). All of their recruited subjects were FAI, while we recruited subjects in general population, which might explain the lower sensitivity and specificity (0.804 and 0.863, respectively) of our study.

The manual ADT is a physical examination routinely used to evaluate the laxity of the ankle joint complexes in patients with CAI because of its good practicability. However, it has been questioned for decades because of subjectivity. Vaseenon et al. [23] found that even though ADT had excellent intraobserver reliability (0.94), the interobserver reliability was only 0.52, which indicated that different examiners were more likely to report inconsistent results when examining the same ankle. Li et al. [7] reported a significant difference in the diagnostic performance of ADT between senior and junior doctors, where the sensitivity was only 5.3% in junior doctors but 39.5% in senior doctors. The diagnostic accuracy of manual ADT heavily relies on examiner’s experience. Manual ADT is unreliable and it fails to detect a considerable proportion of ankle ligament tears [24]. This suggests a need of improved methods to quantify ankle joint laxity.

It is been proposed to use arthrometers to perform ADT quantitatively and objectively [25]. Arthrometry has been investigated by a large amount of studies as a method to quantitatively perform ADT and has shown good to excellent effects in differentiating uninjured ankles and ankles with instability [12, 26–29]. However, even though the amount of studies that reported reliable measures of ankle instability is very high, there are only few groups that assessed or published the clinical application and diagnostic accuracy of the respective measurements in terms of sensitivity and specificity [30]. Further, the diagnostic accuracy of arthrometers is still debatable because of the diversity in studies regarding selection criteria, arthrometric devices and reference standards.

In this study, the selection criteria of subjects were based on recommendations of International Ankle Consortium, which proposed selection criteria with the best available evidence [10]. The inconsistency in participant selection criteria across previous studies presents a potential obstacle to the research of arthrometers’ diagnostic accuracy. Lohrer et al. [6] only recruited CAI subjects, and used manual ADT as a grouping standard to diagnose mechanically unstable ankles. However, manual ADT was already proved to be an unreliable tool in research [27]. Cho et al. [31] included patients with ankle instability who would later undergo ankle arthroscopy for treatment after the assessment. However, there is no indication nor ethical justification to perform ankle arthroscopy in every enrolled subject. Rein et al. [32] proposed to use ultrasound analysis for participant selection. Although ultrasound manifests high diagnostic accuracy, high proficiency is required because the difference between injured and uninjured ankles may be too subtle to detect [33]. Standardized selection criteria enhanced the validity of this study’s findings and improved the understanding of arthrometers’ role in diagnosing CAI.

Types of arthrometric devices also play a role in validating diagnostic accuracy. The Hollis ankle arthrometer and the LigMaster are two most frequently used arthrometers. The Hollis ankle arthrometer is reported to have a high to excellent reliability with ICC values between 0.82 [34] and 0.99 [35]. However, neither sensitivity nor specificity has ever been reported. Meanwhile, there were significant differences between an experienced and unexperienced tester [35]. Furthermore, no correlation was found between arthrometric measurement and radiographic results [36]. The LigMaster has a good to high reliability with ICC values between 0.65 [37] and 0.9 [38]. However, although significant differences between CAI and controls were reported, a study revealed sensitivity values around 0.36 and specificity between 0.72 and 0.94, making its diagnostic accuracy questionable [12]. Our arthrometer had an excellent test–retest reliability. The ICC value of a single measure was 0.897. When using the average of 3 measures, the ICC value increased to 0.963. Therefore, it was practicable to calculate diagnostic accuracy based on our arthrometer’s collected data.

Differences in the selection of reference standards also influence diagnostic accuracy of arthrometers. A systemic review of in vivo arthrometer measurements showed that studies regarding diagnostic accuracy of arthrometers in an in vivo, clinical setting were still limited [30]. In existing literature that calculated diagnostic accuracy (i.e., sensitivity, specificity), researchers chose different reference standards, and the resulting diagnostic accuracy varied greatly. The sensitivity varied from 0.36 to 0.92, and the specificity varied from 0.40 to 0.93 [6, 8, 12, 39]. Currently, reference standards can be divided into two categories: one is statically measured standards that the applied load is fixed, the other is dynamically measured standards that the applied load is continuously increasing [22, 40]. Lohrer et al. [6] used the ratio of applying force from 40 to 60 N and corresponding joint displacement to represent stiffness, and the sensitivity and specificity were 0.81 and 0.93, respectively, with 5.1 N/mm as a cutoff value. However, it should be noticed that this study only differentiated mechanically stable ankles from mechanically unstable ankles in patients primarily diagnosed with FAI without consideration of population with healthy ankles or copers, so it might be inappropriate to diagnose CAI from healthy ankles based on this study. Rosen et al. [12] chose talar tilt angle 29.4° as a reference standard, and the resulting sensitivity and specificity were 0.36 and 0.72, respectively. Croy et al. [8] set two reference standards when assessing the length of ATFL with ultrasound. Applying 125 N of force with an arthrometer, it was found that the sensitivity and specificity were 0.74 and 0.38, respectively, when the reference standard was 2.3 mm or greater, but 0.83 and 0.40, respectively, when the reference standard was 3.7 mm or greater. Wenning et al. [39] utilized sonography-aided arthrometry and revealed that a cutoff value of > 5.4 mm increase in ligament length during stress sonography had sensitivity of 0.92 and specificity of 0.6. According to the literature review above, it may be concluded that dynamic reference standards exhibit better diagnostic accuracy, which aligns with our study. So far, studies focusing on diagnostic accuracy only compared one single reference standard, consequently resulting in a wide variety of results in terms of sensitivity and specificity.

We recognized several limitations of this study. First, we did not include copers in the study. Besides, there was a significant difference in BMI between the control and CAI groups, which might influence the results. The influence was supposed to be limited, however. Vuurberg et al. [41] already demonstrated that BMI in patients with CAI is significantly higher than that of healthy controls. In addition, this statistically significant difference was minimal (mean 22.76 vs 22.11), so the influence of BMI should be limited. Further, another limitation was that we did not make a comparison between injured and uninjured contralateral ankles. Since the standard normal range of arthrometric ADT was yet undefined, an uninjured contralateral ankle might serve the best reference. However, Guerra-Pinto et al. [9] found there was a wide variety in the average mean differences between injured and uninjured ankles, ranging from − 0.9 to 4.1 mm. This variety was due to high heterogenicity in the study design, testing procedures and measuring methods. Therefore, a study with a standardized protocol that compared between injured and uninjured contralateral ankles was needed to make arthrometric ADT more clinically practical.

## Conclusions

In conclusion, the digital arthrometer measurement could quantitively analyze the ankle laxity with high diagnostic accuracy. The load–displacement ratio would be a reliable and promising approach for chronic ankle instability diagnosis. The load–displacement ratio at 10–40 N had a high diagnostic accuracy with sensitivity and specificity of 0.804 and 0.863, respectively, which was suitable to diagnose patients with CAI in clinical settings.

## Data Availability

The datasets used and/or analyzed during the current study are available from the corresponding author on reasonable request.

## Abbreviations

CAI: Chronic ankle instability
MAI: Mechanical ankle instability
FAI: Functional ankle instability
BMI: Body mass index
ATFL: Anterior talofibular ligament
MRI: Magnetic resonance imaging
CAIT: Cumberland ankle instability tool
ROC: Receiver operating characteristic curve
AUC: Area under a curve
SD: Standard deviation
CI: Confidence interval
LDR: Load–displacement ratio
